# Plant photosynthesis in basil (C3) and maize (C4) under different light conditions as basis of an AI-based model for PAM fluorescence/gas-exchange correlation

**DOI:** 10.3389/fpls.2025.1590884

**Published:** 2025-05-19

**Authors:** Isabell Pappert, Stefan Klir, Luca Jokic, Celine Ühlein, Khanh Tran Quoc, Ralf Kaldenhoff

**Affiliations:** ^1^ Department of Applied Plant Sciences, Faculty of Biology, Technical University of Darmstadt, Darmstadt, Germany; ^2^ Laboratory of Adaptive Lighting Systems and Visual Processing, Technical University of Darmstadt, Darmstadt, Germany

**Keywords:** chlorophyll fluorescence, gas exchange, machine learning, photosynthesis prediction, C3/C4 plants

## Abstract

Photosynthetic activity can be monitored using pulse amplitude modulated (PAM) fluorescence or gas exchange. While PAM provides insight into the light-dependent reactions, gas exchange reflects CO_2_ fixation and water balance. Accurate, non-invasive prediction of photosynthetic performance under varying conditions is highly relevant for phenotyping and stress diagnostics. Despite their physiological link, data from both methods do not always correlate. To systematically investigate this relationship, photosynthetic parameters were measured in maize (*Zea mays*, C4) and basil (*Ocimum basilicum*, C3) under different photon densities and spectral compositions. Maize showed the highest CO_2_ assimilation rate of 30.99 ± 1.54 µmol CO_2_/(m²s) under 2000 PAR green light (527 nm), while basil reached 10.56 ± 0.92 µmol CO_2_/(m²s) under red light (630 nm). PAM-derived electron transport rates (ETR) increased with light intensity in a pattern similar to CO_2_ assimilation, but did not reliably reflect its absolute values under all conditions. To improve prediction accuracy, we applied a machine learning model. XGBoost, a gradient-boosted decision tree algorithm, efficiently captures nonlinear interactions between physiological and environmental parameters. It achieved superior performance (R² = 0.847; MSE = 5.24) compared to the Random Forest model. Our model enables accurate photosynthesis prediction from PAM data across light intensities and spectral conditions in both C3 and C4 plants.

## Introduction

Chlorophyll fluorescence analysis by pulse-amplitude modulated (PAM) fluorometry and gas exchange measurements are essential techniques for investigating the mechanisms underlying photosynthesis. The PAM technique allows for a detailed analysis of parameters such as maximum photosynthetic efficiency (Fv/Fm), photochemical efficiency of Photosystem II (ΦPSII), and related terms (Kornyeyev and Holaday, 2008).

Fv/Fm represents the ratio of variable fluorescence (Fv) to maximum fluorescence (Fm) and indicates the maximum efficiency of Photosystem II (PSII) under optimal light conditions. It shows how effectively light energy is converted into chemical energy. ΦPSII is the quantum yield of the photosynthetic electron transport chain under a given light condition. Accordingly, ΦPSII values reflect the competence with which the plant uses absorbed light for electron transport, which ultimately contributes to the synthesis of NADPH and ATP.

While PAM measurements primarily assess the efficiency of energy conversion in the thylakoid membranes, gas exchange measurements directly assess the diffusion of CO_2_ as well as that of water vapor. Key data collected from gas exchange analysis include photosynthetic rate, transpiration rate, and stomatal conductance. These provide information about net carbon gain and stomatal regulation (Long and Bernacchi, 2003). Even though both techniques are related to photosynthesis they do not monitor the same biochemical reaction. PAM focuses on photochemical reactions at the level of PSII, whereas gas exchange reflects the integrated result of downstream metabolic activity, particularly the fixation of carbon through the Calvin cycle. The two methods are functionally linked, as the energy generated in the light reactions drives the dark reactions. Optimal photochemistry is a prerequisite for effective carbon assimilation. Conversely, impaired light reactions can reduce the flux into carbon metabolism. However, discrepancies arise because several intermediate and downstream processes—such as Rubisco activity, mesophyll conductance, or photorespiration—can modulate the link between photochemical and net carbon fixation (Flexas et al., 2012). This decoupling becomes particularly evident under stress conditions such as high light, where PAM fluorescence may overestimate photosynthetic capacity due to ongoing electron transport despite limited CO_2_ fixation (Baker and Rosenqvist, 2004; Flexas and Medrano, 2002; Genty et al., 1989). If a calculation of the relationship between PAM and gas exchange measurements would be feasible, these could significantly reduce the time required for photosynthetic assessments because PAM measurements are quick and less time-consuming in comparison to gas exchange measurements. PAM fluorometry enables fast and non-invasive measurements of photosynthetic activity, with fluorescence parameters such as Fv/Fm or ΦPSII typically measured within seconds to a few minutes. In contrast, gas exchange measurements are often more time-intensive, requiring stabilization of measurement conditions and taking several minutes to up to an hour per sample. Portable PAM fluorometers can be used both in the field as well as laboratory and provide data within a few minutes, whereas gas exchange measurements are more labor-intensive, requiring specialized equipment, which is substantially larger and data observation requires more time (Baker, 2008). In case of a determinable relation between values from PAM and gas exchange, i.e. using ΦPSII to predict the efficiency of CO_2_ fixation, PAM measurements can be utilized to determine photosynthetic rates. Under optimal conditions there is a good correlation between ΦPSII and the CO_2_ assimilation rate as the electrons produced in PSII are mainly used for CO_2_ fixation (Lysenko et al., 2022). This makes PAM fluorescence measurements a meaningful indicator for photosynthetic activity in different plant species (Maxwell and Johnson, 2000) particularly under moderate light intensities. Genty et al., showed that the quantum yield of PSII electron transport correlates well with carbon assimilation when there are no additional active electron sinks. This relationship is more stable under optimal conditions with moderate light (200 to 400 PAR (Genty et al., 1989). It is the basis for using PAM measurements to estimate the rate of CO_2_ fixation. However, it is increasingly evident that these predictions considerably lose accuracy under non-optimal conditions. For example, Genty et al. (1989) also showed that the correlation between ΦPSII and the gas exchange decreases notably at high light intensities (up to 600 PAR) as alternative electron flows are activated that do not contribute to CO_2_ fixation. The measured photosynthetic rates do not match with the PAM predicted assimilation rate, particularly under high light irradiation. It is explained by excess energy in the photosystem that can no longer be used efficiently and flows into alternative protection mechanisms (Genty et al., 1989). Similar results were reported by Flexas and Medrano (2002) under water deficit conditions. Stomatal conductance regulates the entry of CO_2_ into the leaf. It was reduced by water deficiency more than the capacity to transport electrons. Consequently, a high electron transport rate was measured by PAM, even though the gas exchange was severely restricted. This is due to the water stress induced reduction in stomatal conductance which decreases substomatal CO_2_ concentrations. The CO_2_ limitation is apparently not detected by PAM (Flexas and Medrano, 2002). Baker and Rosenqvist (2004) showed that under severe stress, such as high temperature, electron flow rates remain high, while the CO_2_ exchange is severely restricted. Heat damage can disrupt the function of enzymes such as Rubisco while electron transport in Photosystem II is maintained by alternative mechanisms and detected by PAM. Under these conditions, current CO_2_ assimilation is inefficient (Baker and Rosenqvist, 2004). Under conditions of low CO_2_ concentrations or low temperatures, conventional models also fail (Murchie and Lawson, 2013). Here the assimilation rate depends on enzyme-activity rather than electron transport.

In summary, PAM parameter and assimilation rates are decoupled to a certain extent under the conditions exemplarily mentioned above (Murchie and Lawson, 2013). The current photosynthetic activity is overestimated, as alternative electron fluxes or limitations in stromal processes can affect the results (Baker, 2008; Flexas and Medrano, 2002; Genty et al., 1989). Suboptimal conditions include extreme light intensities, water scarcity, heat stress or non-ideal CO_2_ concentrations.

In addition, the predictive ability of PAM measurements for photosynthetic activity relates to the specific physiology of the plant under investigation due to different photosynthesis pathways (von Caemmerer and Furbank, 2003; Farquhar et al., 1980). C3 plants, such as basil, tend to exhibit higher variations when photorespiratory processes are intensified (Yin and Struik, 2009). It occurs more frequently when light intensity is high (>1000 PAR), and CO_2_ availability is low (e.g., 100 ppm). Then a considerable part of the electron transport is directed toward oxygen degradation and can no longer be used for CO_2_ fixation. Foyer and Noctor (2000) provide a detailed explanation of the relationship between electron transport, oxygen degradation and photorespiration, especially under high light intensities and low CO_2_ availability (Foyer and Noctor, 2000).

In contrast, a linear relationship between PSII activity and CO_2_ fixation was initially expected for C4 plants such as maize (Krall and Edwards, 1992). Unlike C3 plants, they utilize a more efficient CO_2_ fixation mechanism characterized by the spatial separation of CO_2_ uptake and fixation in the Calvin cycle (Sage and Zhu, 2011). This leads to an almost complete elimination of photorespiration and to a stronger correlation between the electron transport rate in PSII and the CO_2_ fixation rate. However, under extreme light intensity or stress conditions, such as drought, high temperatures, or nutrient deficiencies, alternative electron fluxes that do not directly contribute to CO_2_ fixation are also activated in C4 plants.

Finally, nitrogen availability plays a significant role in restricted photosynthesis rate prediction by PAM parameters, particularly in C4 plants. Kromdijk et al. (2016) showed that under conditions of high nitrogen supply, both C3 and C4 plants show a stronger correlation between PAM parameters and CO_2_ fixation, as nitrogen is an important factor for photosynthetic capacity and electron transport rates (Kromdijk et al., 2016). Nitrogen deficiency, on the other hand, significantly weakens this correlation, as photosynthetic capacity is limited under suboptimal nutrient conditions. Thus, C4 plants tend to exhibit a stronger correlation under optimal conditions, while C3 plants are more susceptible to decoupling. As a tool for the model setup, we examined the impact of light on the electron transport rate in PSII obtained by PAM, kept temperature, CO_2_ concentration, water supply, as well as humidity constant and measured gas exchange.

Taken together, a mathematical model is required to predict photosynthetic rates by PAM data under non-optimal conditions. Two machine learning tree-algorithms — Random Forest Regressor (Breiman, 2001) and XGBoost (=Extreme Gradient Boosting) Regressor (Chen and Guestrin, 2016) — are further on validated in this research to predict the relationship between chlorophyll fluorescence (PAM) and gas exchange data. Traditional linear or empirical models often fail to capture the nonlinear and multifactorial nature of photosynthetic responses under variable environmental conditions. Machine learning approaches, particularly tree-based algorithms such as Random Forest and XGBoost, are well suited to model such complex interactions and have shown high predictive accuracy in various plant-related applications (Boulesteix et al., 2012; Lysenko et al., 2022; Saleem et al., 2024; Singh et al., 2016, 2023; Varghese et al., 2023; Wu et al., 2022; Zhang et al., 2020). These models were selected since the underlying processes are non-linear and comprise large sets of input features to accurately predict the assimilation rate. Another advantage of tree-based algorithms is the explainability due to the accessible split points of the input data. Almeida et al. (2020) revealed the capability of tree algorithms for plant classification and identifying different vegetative and floral traits out of a set of 16 traits (Almeida et al., 2020). An accuracy of 89% was achieved on average. Additionally, XGBoost has reached in multiple biological research questions a high accuracy and low error rate and is therefore utilized in this paper for further investigation (Babajide Mustapha and Saeed, 2016). To the best of our knowledge, this is the first study to apply explainable tree-based models to predict CO_2_ uptake from fluorescence-based input data in C3 and C4 plants under diverse light spectra.

The goal of this research is to combine PAM and gas exchange data using machine learning algorithms, to achieve more accurate photosynthesis predictions by PAM acquired data under varying light spectra.

## Materials and methods

### Plant growth conditions

Seeds were pre-soaked in water for a duration of 3 hours, and 20 seeds were subsequently placed in 1 L pots for germination. The plants were maintained in a greenhouse with a 12-hour light/dark cycle, a light intensity of 300 µmol/(m²s), a controlled temperature of 21°C, and 68% humidity. Illumination was provided by SANlight LED lamps from 8:00 am to 8:00 pm, ensuring a constant photon flux density of 300 PAR throughout the photo period. This setup allowed for reproducible growth conditions independent of natural daylight. Plants were watered daily at 12:00 pm. To ensure uniform hydration before measurement, all plants received an additional standardized watering of 100 ml on the day before their introduction into the experimental setup. After a 5-day germination period for maize or a 7-day germination period for basil, the seedlings were transferred into 1 L pots with a 14 cm diameter for maize or a 110 ml pot with a 7 cm diameter for basil and grown for 20 days to reach the appropriate age. The basil plants are repotted in pots with a diameter of 12 cm and a volume of 750 ml after 7 days. The plants used to study the influence of plant age on photosynthesis were cultivated under the same conditions for 6 weeks. Shade plants were cultivated under a light intensity of 100 µmol/(m²s), respectively. All measurements and plant cultivation procedures were carried out between March 2023 and September 2024.

### Experimental conditions and illumination

**Figure 1:**
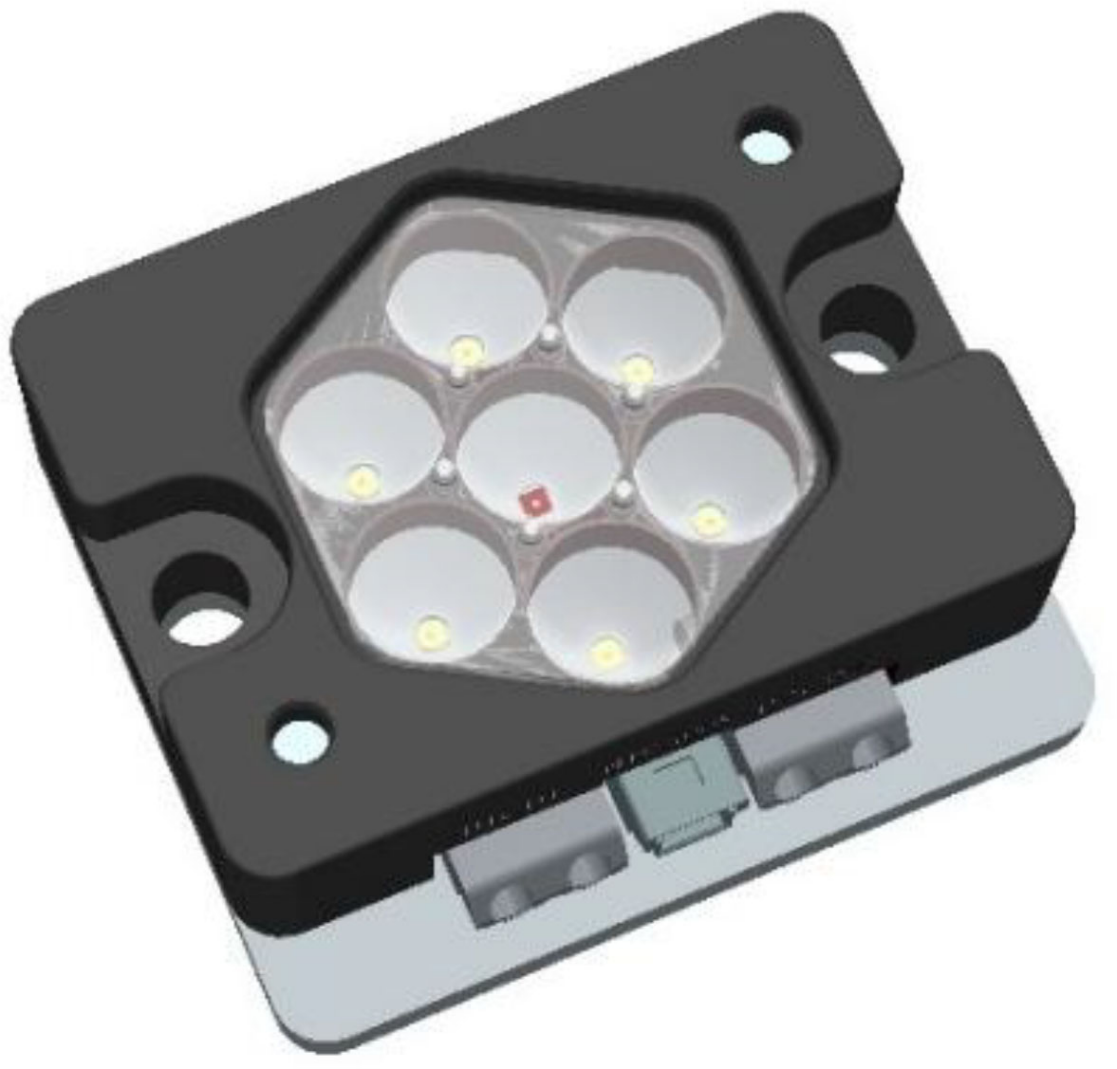
Sevenengine LED modules with total-internal-reflection (TIR) lens technology used for precise illumination in photosynthesis experiments. The modules deliver highly focused light with minimal loss, ensuring efficient targeting of the experimental area (Chips4Light GmbH, Regensburg, Germany).

In this experiment, illumination was provided by Sevengines chips from Chips4Light GmbH (Regensburg, Germany, **Figure 1**), an advanced LED system with a narrow beam angle of ±10°. These state-of-the-art modules utilize total-internal-reflection (TIR) lens technology, which minimizes light losses and delivers exceptional light focus. In contrast to conventional LEDs with standard lenses, the TIR lenses achieve an efficiency of 90% –95%, ensuring that nearly all generated light is directed precisely to the target area.

The LEDs used represent the latest generation of chips, among the brightest available worldwide. The system’s advanced thermal management and optical design guarantee optimal performance for each wavelength. The available modules cover a wide wavelength range between 367 nm and 940 nm, providing flexibility in selecting wavelengths critical for specific experimental requirements.

### Measurements and calculations

#### Steady state gas exchange measurements

Gas exchange was assessed using a portable gas-exchange system (GFS-3000, Heinz Walz GmbH, Germany). Measurement conditions were set to 400 ppm CO_2_, 18000 ppm humidity, a cuvette temperature of 25°C, and light intensities ranging from 0 µmol/(m²s) to 8500 µmol/(m²s). These conditions were chosen to reflect a wide range from darkness to full sunlight conditions, including stress levels under extreme irradiance up to 8500 µmol/(m²s), thereby allowing analysis of both optimal and non-optimal photosynthetic responses (Farquhar et al., 1980). Plants were dark-adapted overnight (16 hours) and then exposed to 100 PAR white light (4000 K) for 75 minutes to initiate photosynthesis before increasing the light intensity. At each light intensity level, plants were allowed to acclimate for 12 minutes to reach a steady-state photosynthetic rate, based on prior observations that this duration is sufficient to stabilize their gas exchange parameters after a change in irradiance. Data from the final 2 minutes of this acclimation period were averaged to ensure steady state conditions before analysis.

#### Steady state chlorophyll fluorescence measurements

Prior to treatment, the plants were dark-adapted for 60 minutes, after which the lowest light intensity (100 PAR) was applied for 60 minutes. Each subsequent light level was applied for 15 minutes, allowing the plants to acclimate and reach a steady state.

Chlorophyll fluorescence was assessed using two PAM fluorometers from Heinz Walz GmbH (Effeltrich, Germany). For image-based measurements, the Imaging-PAM MAXI system (IMAG-MAX/L) was used. Saturation pulses were applied every 30 seconds at a wavelength of 650 nm, with an intensity of 5800 µmol/(m²s) and a pulse duration of 720 ms. For portable measurements, the Junior-PAM fluorometer (CFMG 0225) was employed. Saturation pulses were generated using a 445 nm LED at a typical intensity of approximately 6000 µmol/(m²s). Chlorophyll fluorescence parameters were calculated, including the maximum quantum efficiency of PSII (Fv/Fm), quantum yield of photosynthetic electron transport [ΦPSII = (Fm’ – F)/Fm’], electron transport rate [ETR = ΦPSII * PAR * 0.84 * 0.5], and the quantum yield of energy loss related to non-photochemical quenching [YNPQ = (F/Fm’) – (F/Fm)] from the Chl fluorescence measurements.

### Measurement repetitions

A minimum of ten plants were tested per condition for all chlorophyll fluorescence and gas exchange experiments. When multiple conditions were assessed on the same plant, the order of these conditions was randomized. In total, 360 plants were analyzed in this study.

### Machine learning

Two machine learning models, Random Forest and XGBoost, were adapted to link chlorophyll fluorescence measurements to plant gas exchange measurements. These tree-based models were selected due to their robustness in handling non-linear relationships, high interpretability, and successful application in plant physiological modeling (Singh et al., 2023; Zhang et al., 2020).

The hyperparameters of the utilized models are optimized to enhance their predictive performance. The Randomized Search Cross-Validation (Bergstra and Bengio, 2012) technique was implemented to improve the hyperparameters. Due to the high number of interacting features, randomized hyperparameter tuning was used to avoid overfitting and ensure generalizability of the models. This approach employs a random selection process to choose hyperparameter combinations and subsequently performs cross-validation. The optimized hyperparameter types for the Random Forest Regressor are: the number of trees in the forest (*n_estimators*), the maximum number of features considered for splitting at each node (*max_features*), the maximum depth of the tree (*max_depth*), the minimum number of samples required to split an internal node (*min_samples_split*), and the minimum number of samples required to be at a leaf node (*min_samples_leaf*). We implemented the Random Forest model and conducted 100 iterations, sampling the specified hyperparameters. The following search ranges were applied:

- *n_estimators* = [100,200,300,400,500,800,1000],
- *max_features* = [None, “sqrt”, “log2”],
- [5 <= *max_depth* <= 20] with step size 1,
- [0.1 <= *min_samples_split* <= 0.3] with step size 0.02 and.
- [0.05 <= *min_samples_leaf* <= 0.2] with step size 0.02.

for the Random Forest Regressor. The hyperparameter search interval for the XGBoost Regressor are:

- *n_estimators* = [100, 200, 350, 500, 600, 700, 1000],
- *max_depth* = [4, 6, 8, 10, 12],
- *learning_rate* = [0.01, 0.1],
- *min_child_weight* = [50, 100, 150, 200, 250, 300, 350],
- *gamma* = [100, 200, 500, 1000, 1500, 2000, 2500, 3000, 3500] and
- *colsample_bytree* = [0.8, 0.9, 1.0].

Whereas the the *learning_rate* specifies the step size shrinkage used in updates to prevent overfitting, the *min_child_weight* describe the minimum sum of instance hessian-weight needed in a child, *gamma* is the minimum loss reduction required to make a further partition on a leaf node of the tree and *colsample_bytree* is a family of parameters for subsampling of columns.

To provide a reliable evaluation with hyperparameter optimization a 5-fold cross-validation was employed.

## Results

The machine learning model input parameters must comprise factors with significant impact on photosynthesis rates, as these are critical for generating accurate predictions. It comprises environmental conditions such as photon density, temperature, and humidity, as well as plant-specific traits such as species, developmental stage, or leaf position, all contributing to the overall photosynthetic performance. The following sections are structured in two parts: The first part (Input-Parameter) elucidates the need and the significance of the features whereas the second part (Machine Learning) employs data of these features in the machine learning models to predict the assimilation rate by Random Forest or XGBoost.

### Input-parameter

#### Cultivation under different light conditions

**Figure 2:**
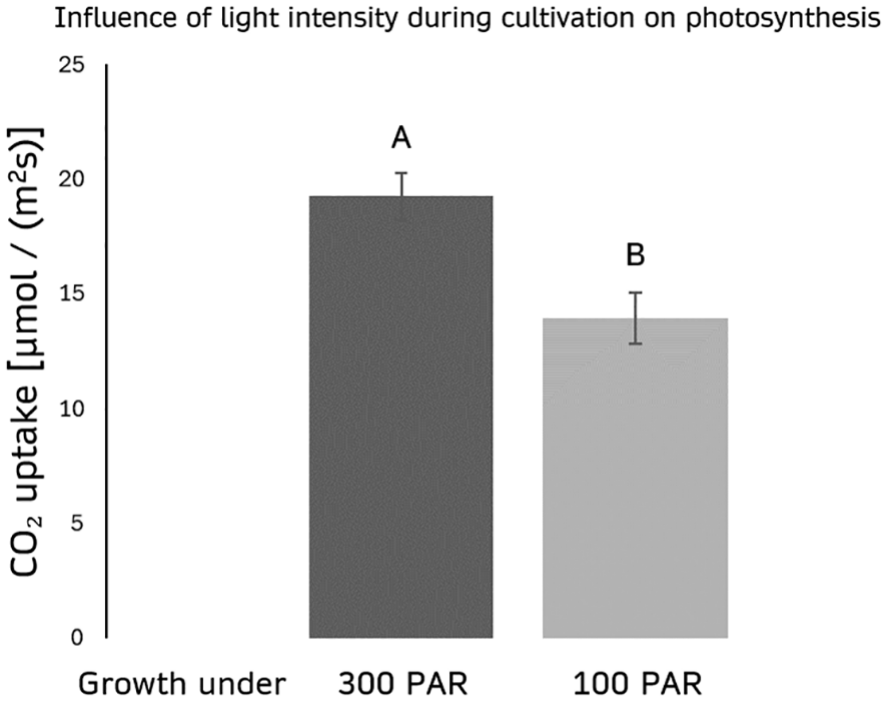
Effect of light intensity during cultivation on photosynthesis. The figure illustrates the assimilation rate (µmol/(m²s)) at 2000 PAR white light (4000 K) of 21-day old maize plants grown under two different light intensities: 300 PAR and 100 PAR. n=10 for each treatment. Error bars indicate standard deviation of the measurements. p(A;B) < 0,01.

Cultivation of plants under varying light intensities results in the development of so-called sun and shade plants, with distinct physiological characteristics. When maize was exposed to 2000 PAR, plants grown under 300 PAR white light (3500K) showed an assimilation rate of 19.7 ± 1.007 µmol/(m²s) whereas plants grown under 100 PAR white light exhibit a significantly lower assimilation rate of 13.9 ± 1.10 µmol/(m²s) under the same conditions (**Figure 2**).

Sun leaves, cultivated under high light intensities, exhibit significantly higher CO_2_ assimilation rates compared to shade leaves, which developed under lower light intensities. This disparity in assimilation rates under identical light conditions highlights the critical influence of light intensity during cultivation. Therefore, it is essential to include light intensity during plant growth as a variable in any model predicting CO_2_ assimilation, as it directly impacts the current photosynthetic performance of sun or shade leaves even if data were obtained under significantly higher light irradiation than those used for plant cultivation.

#### Plant species: diverse pathways and their impact

Incorporating both C3 and C4 plants into a model examining the relationship between PAM and gas exchange is supportive for capturing a broader range of photosynthetic responses across different environmental conditions. C3 plants, such as basil, rely on the Calvin cycle for carbon fixation, where CO_2_ is directly incorporated into a three-carbon compound. These plants perform better under cooler, moist conditions with moderate light. In contrast, C4 plants, such as maize, utilize a specialized carbon fixation pathway that concentrates CO_2_ in specific cells, reducing photorespiration. This adaptation makes C4 plants more efficient under high light intensity, elevated temperatures, and dry conditions.

**Figure 3:**
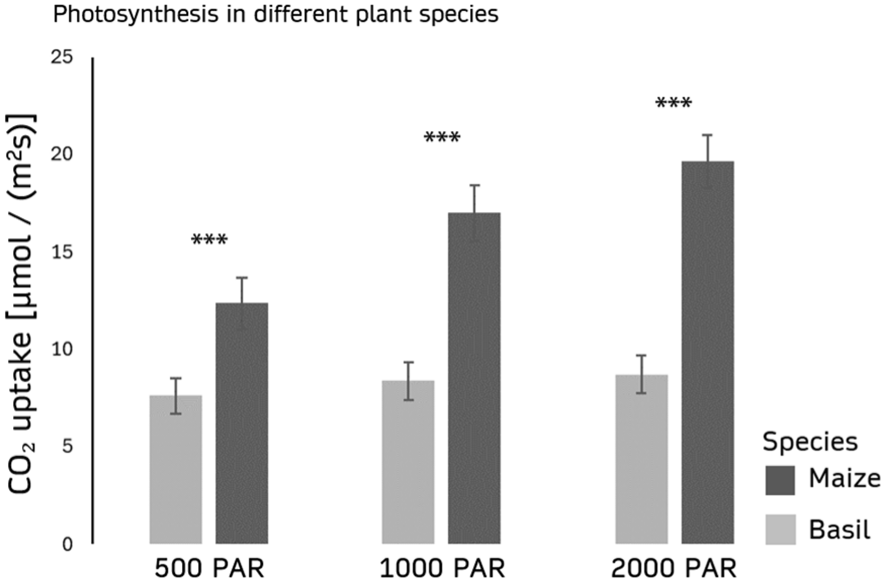
Photosynthesis in different plant species under varying light intensities. The graph compares the assimilation rate (µmol/(m²s)) of the C3 plant basil and the C4 plant maize under light intensities of 500 PAR, 1000 PAR, and 2000 PAR (White light, 4000 K). n=10 for each treatment. Error bars indicate standard deviation of the measurements. p(Maize;Basil) < 0,001. *** indicates a statistically significant difference between species at p < 0.001.

The photosynthetic rates of maize (C4) and basil (C3) under varying light intensities (PAR, white light) clearly reflect their distinct photosynthetic efficiencies. At 500 PAR, maize exhibits an assimilation rate of 12.35 ± 1.309 µmol/(m²s), while basil reaches 7.613 ± 0.916 µmol/(m²s). As light intensity increases to 1000 PAR, maize achieves 16.981 ± 1.439 µmol/(m²s) compared to basil’s 8.376 ± 0.961 µmol/(m²s). At the highest light intensity (2000 PAR), maize reaches 19.635 ± 1.362 µmol/(m²s), whereas basil only shows a slight increase to 8.702 ± 0.969 µmol/(m²s) (**Figure 3**).

By including data from both C3 and C4 plants, the model can more accurately simulate photosynthetic responses under varying environmental conditions. This broader approach enables a more comprehensive understanding of the factors influencing the relationship between PAM measurements and gas exchange, accounting for the distinct physiological strategies of these plant types.

#### Plant age: evaluating its impact on photosynthetic rate

We conducted experiments to assess the effect of maize plant age on photosynthesis rates. To ensure consistency, photosynthesis was measured at the same location for six consecutive weeks.

**Figure 4:**
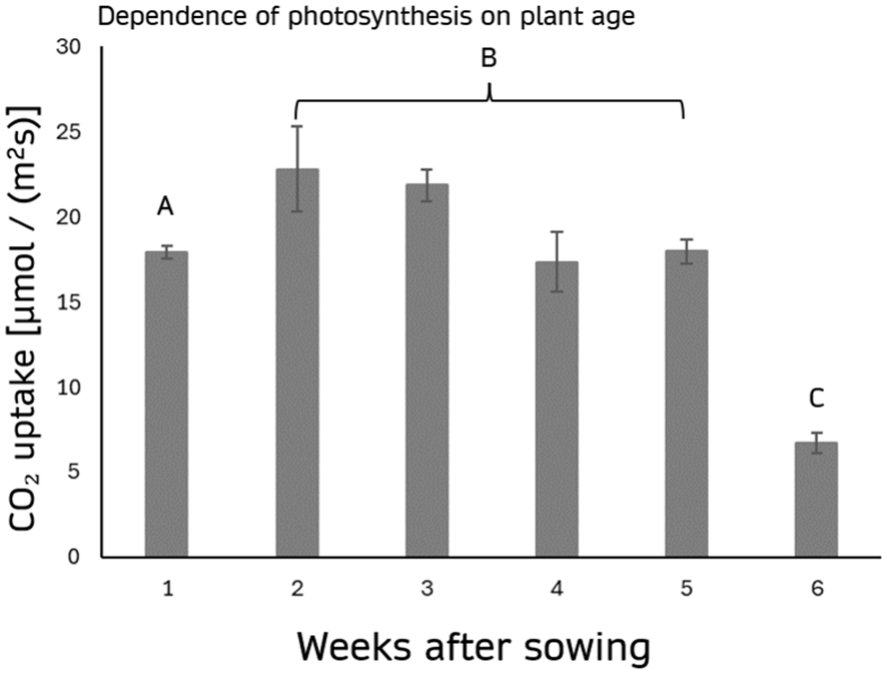
Dependence of photosynthesis on plant age. The graph depicts the assimilation rate (µmol/(m²s)) under 2000 PAR (white light, 4000 K), measured weekly from the first to the sixth week after sowing. The highest rates are observed in the second and third weeks, with a marked decline in the following weeks, reaching the lowest value by the sixth week. A, B, and C indicate the significance groups. The error bars represent the standard deviation of the measurements. Groups A, B, and C differ significantly (p < 0.001).

The assimilation rates increased from week 1 (17.937 ± 0.376 µmol/(m²s)) to a peak in week 2 (22.805 ± 2.480 µmol/(m²s)) and week 3 (21.862 ± 0.942 µmol/(m²s)), before gradually declining in week 4 (17.337 ± 1.767 µmol/(m²s)) and week 5 (17.972 ± 0.721 µmol/(m²s)). Notably, a significant reduction in assimilation rates was observed in week 6 (6.695 ± 0.579 µmol/(m²s)), marking a sharp decline in photosynthesis rates (**Figure 4**).

This suggests that while photosynthesis rates remain relatively stable during early developmental stages a notable reduction occurs as the plant matures beyond the fifth week.

#### Measurement location: influencing photosynthetic performance

**Figure 5:**
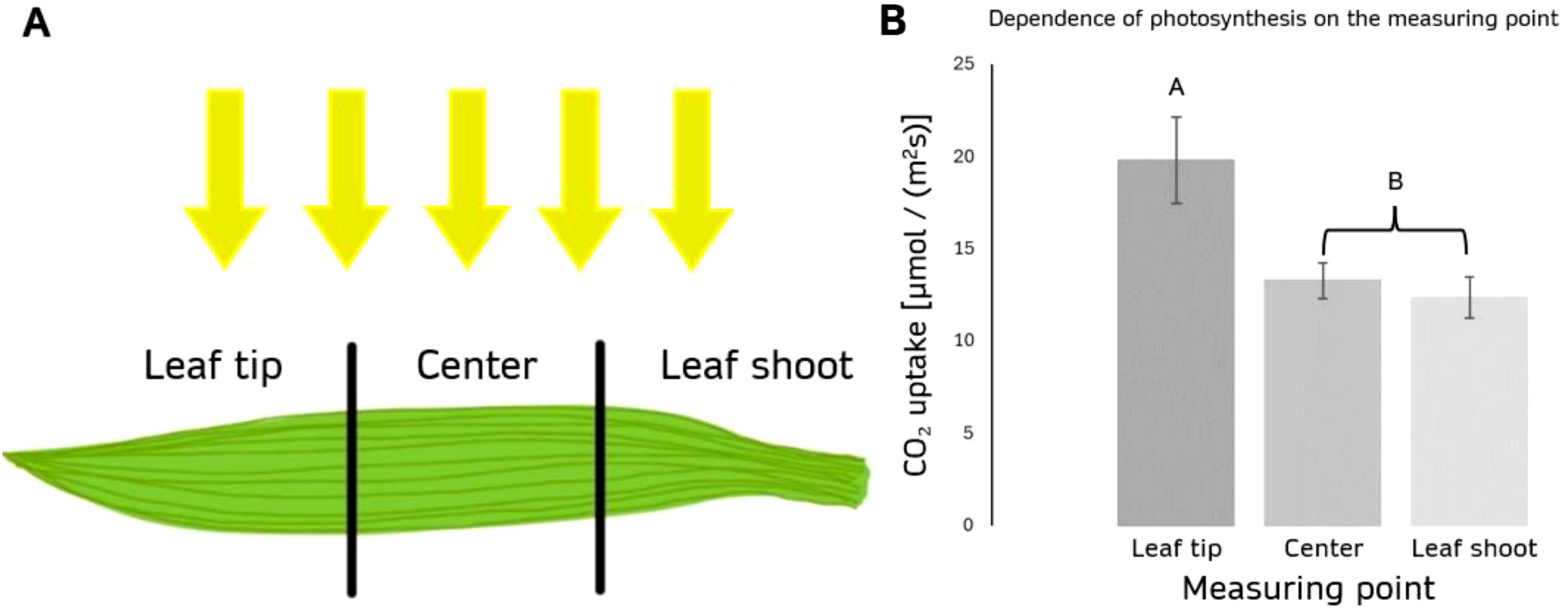
Dependence of photosynthesis on the measuring point of 3 weeks old maize plants. **(A)** Schematic representation of the three analyzed leaf segments relative to the light source during maize cultivation. **(B)** The graph illustrates the assimilation rate (µmol/(m²s)) under 2000 PAR (white light, 4000 K), measured at three locations: the tip, center, and leaf base. n=10 for each treatment. The error bars indicate the standard deviation of the measurements. p(Leaf;Center) < 0.01, p(Leaf;Shoot) < 0.01, p(Shoot;Center) = 0.534. Groups A and B differ significantly (p < 0.001).

The photosynthetic rate is not uniform across different locations in the leaf or plant. Our data indicate that older leaf tissues exhibit higher photosynthetic rates compared to younger leaf areas. Measurements taken at different locations on the maize plant (**Figure 5A**) reveal significant variation. The photosynthetic rate at the leaf tip was 19.760 ± 2.337 µmol/(m²s), while the center of the leaf showed a lower rate of 13.251 ± 0.990 µmol/(m²s), and the base had the lowest rate of 12.322 ± 1.108 µmol/(m²s) (**Figure 5B**). This variability suggests that age-related changes within individual leaves influence photosynthetic performance, with older tissues being more efficient in carbon assimilation. Consequently, spatial differences across the leaf must be considered when assessing and comparing photosynthetic rates of different plants, as tissue age can significantly impact the overall measurement.

#### Photosynthesis as a function of leaf position

**Figure 6:**
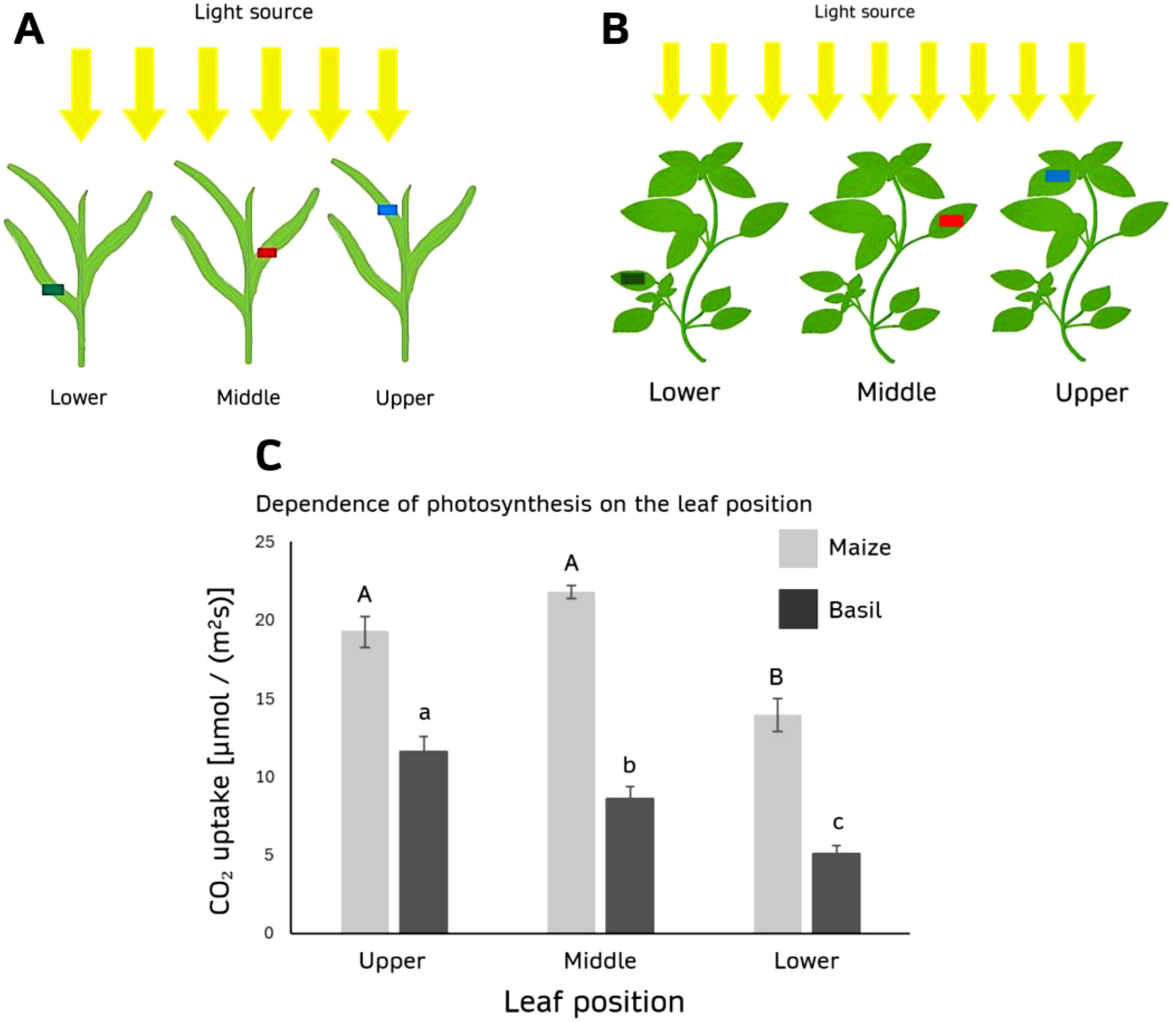
Dependence of photosynthesis on the leaf position of 3 weeks old plants. **(A)** Schematic representation of the three analyzed leaf positions relative to the light source during maize cultivation and **(B)** during basil cultivation. **(C)** The graph illustrates the assimilation rate (µmol/(m²s)) under 2000 PAR (white light, 4000 K), measured at three leaf positions: the lowest, middle, and upper for basil and maize. Maize: p(upper; middle) = 0.015, p(upper; lower) = 0.003, p(middle; lower) < 0.001. Groups A and B differ significantly (p < 0.001). Basil: Groups a, b, and c differ significantly (p < 0.001).

The photosynthetic rate is also not uniform along the shoot axis in different leaf layers of a single plant, and this variability is species dependent (**Figures 6A, B**).

For maize, the middle leaf-layer exhibited the highest photosynthetic rate at 21.795 ± 0.421 µmol/(m²s), followed by the upper layer at 19.247 ± 1.004 µmol/(m²s), with no significant difference between these two layers. The lowest photosynthetic rate was observed in the lower leaf layer, with a mean rate of 13.941 ± 1.050 µmol/(m²s).

In contrast, basil showed a different pattern, with the upper leaf layer having the highest photosynthetic rate at 11.618 ± 0.923 µmol/(m²s), followed by the middle layer at 8.614 ± 0.760 µmol/(m²s). The lower leaf layer exhibited the lowest rate, with 5.104 ± 0.513 µmol/(m²s) (**Figure 6C**). This data highlights species-specific differences in the spatial distribution of photosynthetic activity within the canopy.

#### Photosynthetic rates of corn and basil under various intensities of white and monochromatic light

**Figure 7:**
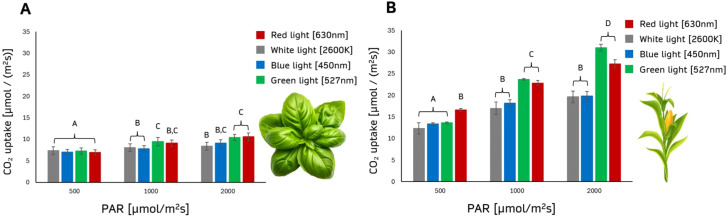
Photosynthesis depends on light intensity and wavelength. The chart shows plant assimilation (µmol/(m²s)) under different light intensities and wavelengths for Basil **(A)** and Maize **(B)**. PAR levels of photosynthetic radiation were 500, 1000, and 2000 µmol/(m²s). Light treatments included red light (630 nm), white light (2600 K), blue light (450 nm), and green light (527 nm). n=10 for each treatment. For a PAR of 500 µmol/(m²s), assimilation rates were similar across all light treatments. Groups A, B, C and D differ significantly (p < 0.001).

We collected data to analyze the photosynthetic rates of maize (*Zea mays*) and basil (*Ocimum basilicum*) under different intensities of white light and monochromatic light (**Figure 7**). We investigated 500 PAR, 1000 PAR, and 2000 PAR under white (2600 K), red (630 nm), blue (450 nm), and green light (527 nm).

At a PAR of 500 µmol/(m²s), the photosynthetic rates of basil were relatively consistent across all light treatments, while maize showed the highest photosynthetic rate under red light (16.620 µmol/(m²s)), which was notably higher than the rates under blue, green, or white light. At 1000 µmol/(m²s), basil exhibited higher rates under red and green light, with the red light reaching 9.076 µmol/(m²s), compared to lower rates under blue and white light. Similarly, maize showed improved assimilation under green light (23.655 µmol/(m²s)) and red light, which were both higher than the blue and white light conditions. At the highest light intensity of 2000 µmol/(m²s), basil continued to show enhanced photosynthetic rates under red and green light, with the highest rate of 10.557 µmol/(m²s) under red light. For maize, green light produced the highest photosynthetic rate (30.992 µmol/(m²s)), followed closely by red light, with both outperforming blue and white light significantly.

Therefore, it is essential to incorporate both light spectrum and light intensity as important variables in the predictive model, as they significantly influence photosynthetic performance and thus impact the accuracy of CO_2_ assimilation rate predictions.

### Feature definition

To establish a connection between chlorophyll fluorescence and gas exchange parameters, machine learning algorithms were applied. These algorithms operate on datasets composed of individual features (**Table 1**).

**Table 1:** Overview of the features used for model training and evaluation, including environmental conditions, physiological parameters, and spectral components.

Feature	Meaning
par_total	Total light intensity (400–730 nm), measured in µmol/(m²s).
2600K, 3200K, 4000K	Light intensity contributed by the respective white light (2600K, 3200K, 4000K), measured in µmol/(m²s).
405_nm – 730_nm	Light intensity contributed by the respective monochromatic light at each wavelength, measured in µmol/(m²s).
plant age	Age of the plant since sowing, measured in days.
plant_id	Unique sequential identifier assigned to each plant to ensure traceability of measurements.
month	The month in which the measurements were conducted.
year	The year in which the measurements were conducted.
e	Transpiration rate measured using the gas exchange system, expressed in mmol H_2_O/(m²s).
g	Stomatal conductance to water vapor, measured in µmol H_2_O/(m²s).
tleaf	Leaf temperature measured using the gas exchange system, expressed in °C.
Y(II), ETR, NPQ	Quantum yield of photosystem II, Electron transport rate, Non photochemical quenching, measured using the PAM fluorometer.
leaf_level	Indicates the leaf layer within the plant where the measurements were conducted.
m_segment	Specifies the measured segment of the leaf: proximal (near the stem), middle, or distal (leaf tip).
species	Categorical variable indicating whether the plant is *Zea mays* (C4) or *Ocimum basilicum* (C3)

#### Machine learning - input feature set

The input data processing for the tree algorithms were structured into the following four steps: (1) data cleaning, (2) data transformation, (3) model training and (4) model evaluation.

Initially, in the data cleaning step the input dataset is prepared for further evaluation. First the non-numeric features that were only provided for the human description of the data were removed. This includes the textual described condition and the name of the light source. Furthermore, duplicated data as well as rows which contain missing values were dropped (Lee et al., 2021).

In the remaining data each feature with less than three distinct values was also removed since this attribute would provide only little valuable information (Ilyas and Chu, 2019). In this stage, the initial dataset of 1783 data with 36 features was optimized to 1739 and 26 features.

Following the initial data preparation, additional functions were developed in the second step - the data transformation - to improve the strength of the prediction models. This included the creation of additional 20 interaction features, to map the domain knowledge of the researchers into data. This was achieved by combining the features for specifying the light with the recorded intensities per wavelength in pairs (Hornung and Boulesteix, 2022). The following wavelengths were considered: 405 nm, 430 nm, 450 nm, 465 nm, 485 nm, 500 nm, 527 nm, 550 nm, 590 nm, 630 nm, 660 nm, 730 nm and are pairwise multiplied. By capturing the precise interaction between two wavelengths, the model can gain a better understanding, that these features are related and can thus make more accurate predictions (Hornung and Boulesteix, 2022). Furthermore, the depth of the tree can be reduced since this correlation must not be learned by the tree.

As the evaluation date is a non-numeric feature, a new feature was created by counting the months from the year of the first measurement with the equation: ((year – first_year)*12 + month) (Heaton, 2016). This was necessary to analyze the time dependencies and seasonal patterns in greater detail. In combination with the month the temporal course and seasonal fluctuations that could affect the physiological processes in a plant are considered. Since tree-based algorithms will be utilized as models the absolute features were further processed and no standardization or normalization process is required (Breiman, 2001).

Several feature sets were created utilizing different models to determine the optimal set of input parameters for the model. During the analysis, the development of so called minimal and interaction-based feature sets are considered and are subsequently explained.

The minimal feature set comprised a core selection of characteristics believed to be relevant. The crucial feature subset comprised Y(II), ETR, par_total and plant age. The Interaction data set extends the Minimal set by incorporating additional variables, including plant_id, month and year. Furthermore, specific spectral light components (430 nm, 450 nm, 485 nm, 500 nm, 527 nm, 550 nm, 590 nm, 630 nm, 660 nm, as well as various white light sources at 2600 K, 3200 K, and 4000 K) are considered. To enhance predictability, a feature set is developed based on interactions between two wavelengths at a time as explained before. Additional factors also include tleaf, e, gH_2_O, leaf_level, and m_segment.

Using these two different sets, the influence of diverse combinations of features on the model was systematically evaluated. The minimal set provided insight into the basic and essential predictors, while the interaction term-based set focused on capturing high-order complex interactions and determining their additional value and included all provided features.

These features are further evaluated by two machine learning tree-algorithms: Random Forest Regressor (Breiman, 2001) and XGBoost Regressor (Chen and Guestrin, 2016).

The utilized data set was split into two subsets for training and testing with 80% train and 20% test data. The divide assures that the model’s accuracy and ability to generalize is determined by evaluating its performance on data that were not used for training. This ensures that the performance of the trained models is evaluated on data that has not been previously observed during the training phase, thereby yielding a transferable and generalized model.

#### Machine learning - hyperparameter search

The previous section stated the importance of light intensity, plant age, measuring point, leaf position as well as different wavelengths. Further on, the results of the tree-based machine learning models XGBoost and Random Forest are stated. Overall, 1739 data points with a maximum of 46 features are utilized as input.

The goal of this model is to achieve the highest level of performance as well as a minimal difference in the training and test data set for predicting the assimilation rate based on the input features. As a first step of the model creation process, the impact of modifications in a solitary hyperparameter on the accuracy of the model were closely examined. The model performance was assessed involving two primary metrics: the Mean Squared Error (MSE) and the R-squared (R²) Score whereas the R² Score is the main metric.

**Figure 8:**
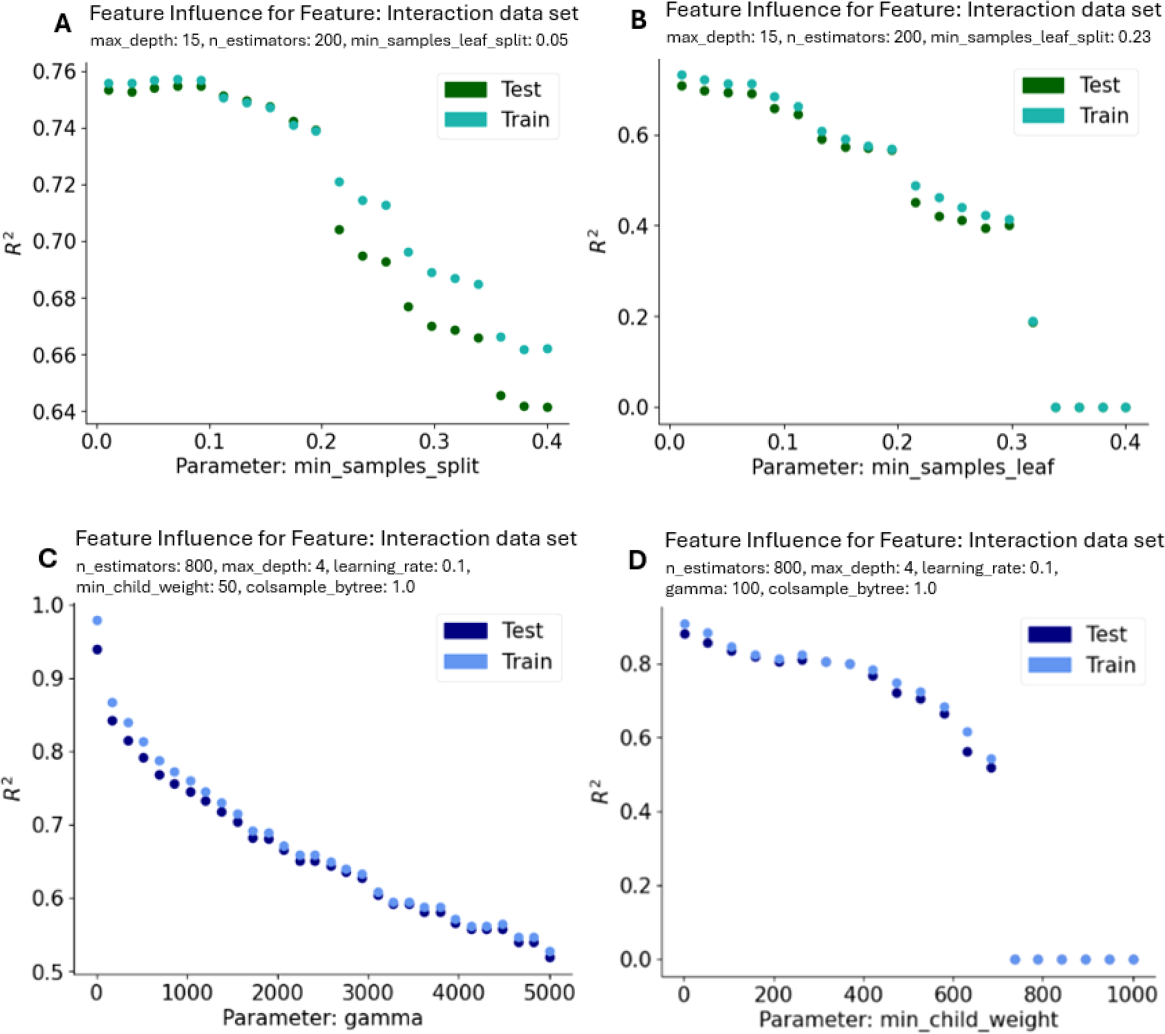
Evaluation of two parameters of the Random Forest Regressor **(A, B)** and two parameters for XGBoost **(C, D)** with the largest spread and decrease between training and test data set with regards to the R-squared Score R². All diagrams show the interaction (X_interactions) data set. A similar course is revealed for the minimal data set. The used non-default values are displayed on the top of each plot.

The mentioned hyperparameter ranges were therefore obtained by a visual evaluation of these parameters over the R-squared (R²) score. A selected number of plots can be inspected in **Figure 8**. These plots were selected since they illustrate the greatest change over the parameter in the R² Score. For a generalized model the R² difference between the train and test data set must be minimized.

The final parameters for Random Forest are:

- Interaction data set:
  - max_depth=6, min_sample_leafs=0.05, min_sample_split=0.1, n_estimators=800.
- Minimal data set:
  - max_depth=6, min_sample_leafs=0.05, min_sample_split=0.1, n_estimators=800.

and the parameters for XGBoost are:

- Interaction data set:
  - colsamples_bytree=0.9, gamma=100, learning_rate=0.1, max_depth=4, min_child_weight=50, n_estimators=500.
- Minimal data set:
  - colsamples_bytree=1.0, gamma=100, learning_rate=0.1, max_depth=8, min_child_weight=50, n_estimators=600.

#### Machine learning – feature importance

**Figure 9:**
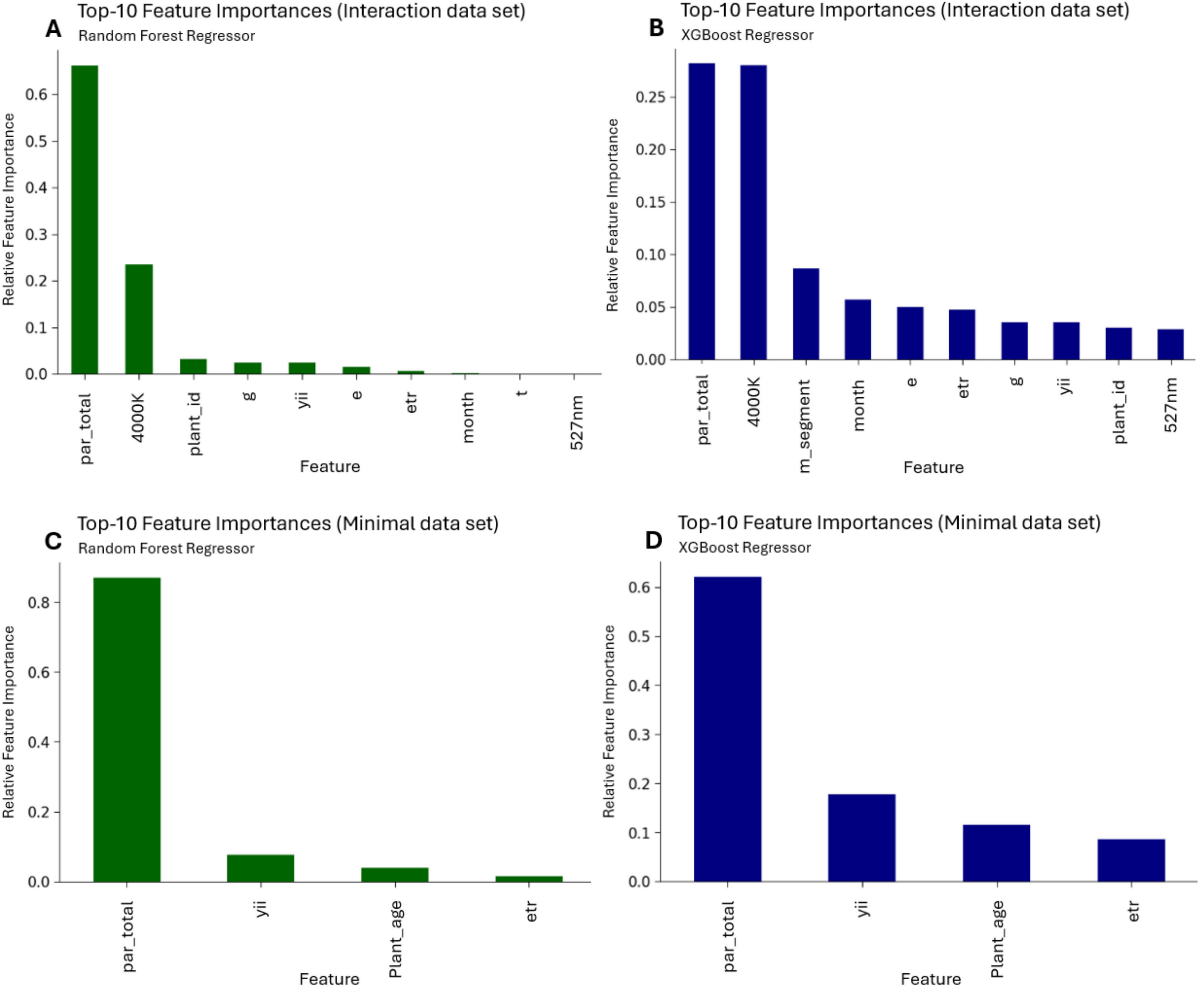
Random Forest Regressor on the left plots **(A, C)** and XGBoost Regressor in the right plots **(B, D)**. The upper plots A and B reveal the feature importance for the interaction (X_interactions) data set whereas the lower plots C and D the minimal (X_min) data set represent.

In addition to assessing the overall performance of the model, it is crucial to evaluate the significance of a feature in predicting the target variable. The analysis in **Figure 9** highlights the key features that have a significant impact on the model’s predictions, offers insight into their biological interpretation and were obtained by the best model after hyperparameter optimization. The importance of a feature in the model indicates its contribution to the final prediction and the sum of the relative importance is 1.0.

From **Figure 9** and the interaction data set the features g, e, par_total, etr and yii have the highest importance, whereas in the minimal data set likewise par_total, etr and yii are revealed to have the highest impact on the model to predict the chlorophyll fluorescence measurements.

#### Machine learning – model performance

**Figure 10:**
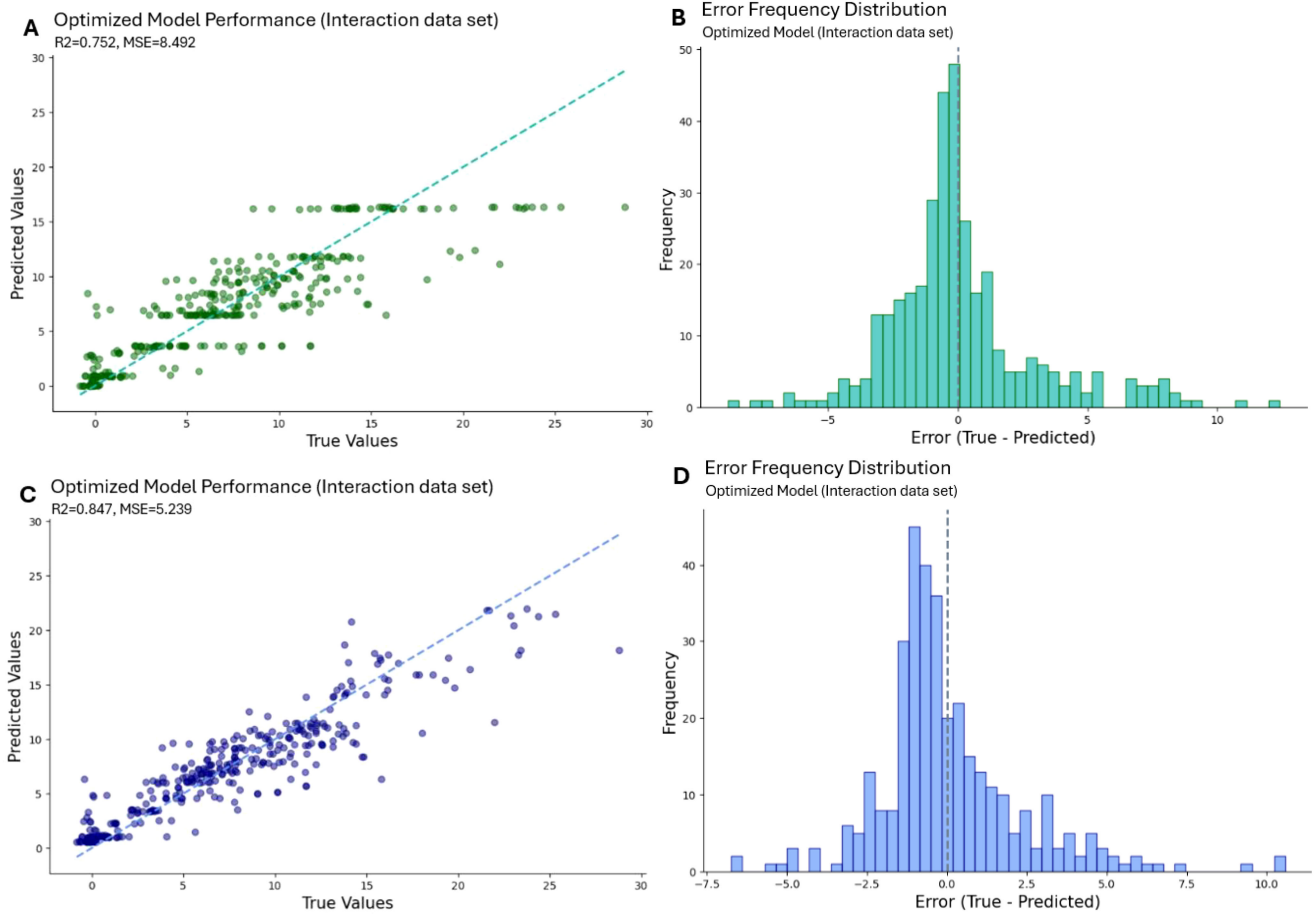
**(A, B)** illustrate the results of the Random Forest algorithm whereas **(C, D)** shows the XGBoost algorithm. In **(A, C)**, the predicted values are revealed over the true values from the measurement. **(B, D)** show the absolute error as a histogram.

The model performance results for Random Forest (A and B) and XGBoost (C and D) algorithms are evaluated based on predicted vs. true values scatter plots and error frequency distributions (**Figure 10**).

The scatter plot indicates that the Random Forest (**Figure 10A**) demonstrates a correlation between predicted and true values. The dispersion around the regression line is considerable and occasionally reaches a maximum for elevated assimilation rates (>15 µmol/(m²s)). The R² score of 0.752, and an MSE of 8.5 indicates favorable outcomes for the test dataset. Nevertheless, the fitted values for elevated assimilation rates tend to be slightly underestimated, resulting in systematic discrepancies. The error frequency figure (**Figure 10B**) exhibits a somewhat centralized distribution with a peak near 0, indicating that the model predominantly yields unbiased predictions for most observations. Nevertheless, the distribution of errors is extensive, particularly on the positive side, suggesting occasional underestimation of higher values.

The scatter plot for the XGBoost model (**Figure 10C**) demonstrates a significantly tighter clustering around the regression line, particularly for values below 20 µmol/(m²s). The enhanced alignment is evidenced by the elevated R² score of 0.847 and the reduced MSE of 5.24 for the test dataset. The variances are reduced, and the model predictions align more closely with the true values throughout the range. Most of the errors are concentrated near zero, signifying that this model more successfully minimizes both bias and variation (**Figure 10D**).

XGBoost surpasses Random Forest in both datasets, producing superior R² scores and reduced MSE values (**Table 2**).

**Table 2:** The results for the two tree-algorithms Random Forest and XGBoost with the R2 score as well as with the MSE.

Dataset	Random Forest		XGBoost	
	Train R^2^ (MSE)	Test R^2^ (MSE)	Train R^2^ (MSE)	Test R^2^ (MSE)
**minimal data set**	0.737 (8.94)	0.741 (9.45)	**0.776 (8.64)**	**0.762 (8.22)**
**interaction data set**	0.756 (8.31)	0.752 (8.5)	**0.87 (4.44)**	**0.847 (5.24)**

Bold values highlight the best test performance (highest R^2^, lowest MSE) for each dataset.

## Discussion

### Photosynthesis key insights from feature importance analysis

Light intensity is the dominant factor influencing photosynthetic assimilation rates in both datasets. In the minimal dataset, it accounts for over 60% of the total importance, while in the interaction-based dataset, it remains among the most significant features, particularly in combination with 4000 K. This is consistent with the fundamental role of light as the energy source driving photosynthesis, initiating chlorophyll excitation, electron transport, and carbon fixation (Genty and Harbinson, 2004).

The importance of spectral properties further underscores that photosynthesis is not solely governed by light quantity but also by spectral quality. The spectral composition influences chlorophyll and accessory pigment absorption, light capture efficiency, and energy conversion (Simkin et al., 2022). This highlights the necessity of incorporating both total light intensity and spectral data into predictive models to enhance accuracy.

Chlorophyll fluorescence parameters, particularly Y(II) and ETR, also emerge as key predictors. Y(II) reflects the photochemical efficiency of Photosystem II (PSII), while ETR represents electron transport activity, making them critical indicators of photosynthetic function under varying light conditions (Yousef et al., 2021). Their prominence underscores the value of fluorescence-based metrics in assessing the physiological state of the photosynthetic apparatus (Kalaji et al., 2016).

In the interaction-based dataset, 4000 K and specific wavelength interactions, such as 527 nm, further emphasize the role of spectral composition. The significance of these variables suggests that certain wavelengths enhance assimilation efficiency, supporting the notion that different pigment systems selectively respond to specific spectral ranges (Kuleshova et al., 2018). Notably, green light (e.g., 527 nm) is not merely reflected but actively contributes to photosynthesis, challenging traditional assumptions about its limited role (Arsenault et al., 2020; Brodersen and Vogelmann, 2010; Chen et al., 2024; Evans and Anderson, 1987; Kaiser et al., 2019; Nishio, 2000; Smith et al., 2017; Sun et al., 1998; Terashima et al., 2009; Virtanen et al., 2022).

Plant-specific traits, including plant age and leaf segment (m_segment), also play a role in both models. Plant age is particularly relevant in the minimal dataset, indicating age-related variations in photosynthetic capacity. This confirms that photosynthesis is not static but changes over a plant’s lifespan. The importance of m_segment suggests that photosynthetic efficiency varies within individual leaves, with older tissues generally exhibiting higher assimilation rates than younger ones (Dwyer and Stewart, 1986; Jahan et al., 2023; Pick et al., 2011; Prochazkova et al., 2001; Suzuki et al., 1987; Trouwborst et al., 2011). Consequently, spatial variation within leaves must be considered in photosynthesis measurements to avoid confounding effects (Kutík et al., 2001). A more physiological level of interpretation is provided by comparing the intrinsic photosynthetic pathways of C3 and C4 species. Maize, a C4 species, possesses a CO_2_-concentrating mechanism that minimizes photorespiration and results in a more linear, efficient relationship between light-driven electron transport (as assessed by PAM) and net CO_2_ assimilation (Hatch, 1987). In contrast, C3 plants like basil show a less direct correlation between these two processes, especially at high light, due to higher photorespiratory losses (Lee and Kim, 2024). Despite the described differences in the photosynthetic pathways of the studied species, the feature indicating the photosynthetic type (C3 vs. C4) does not appear among the top ten predictors of assimilation rate in the machine learning model.

### XGBoost’s superiority in capturing complex biological interactions

The XGBoost model outperforms Random Forest in predicting photosynthetic assimilation rates, achieving a higher R² score of 0.847 and a lower mean squared error (MSE) of 5.24. These results indicate that XGBoost captures a greater proportion of the variability in photosynthesis rates and provides more accurate predictions. The model did not regard the interaction features being significant. Only particular spectral components, specifically 4000 K and 527 nm, were recognized as significantly relevant. This highlights that individual wavelengths, rather than complex spectrum relationships, directly affect photosynthetic performance.

A key advantage of XGBoost lies in its ability to model complex feature interactions, particularly those associated with specific wavelengths, which may be overlooked by Random Forest. This is crucial, as photosynthesis involves nonlinear relationships between light, temperature, gas exchange parameters, and plant-specific traits. The ability of XGBoost to integrate diverse features, including par_total, chlorophyll fluorescence parameters, spectral data, and plant-specific characteristics, ensures biologically meaningful predictions closely aligned with actual photosynthetic processes.

Additionally, XGBoost effectively manages feature interactions, reducing both bias and variance, as evidenced by its tighter distribution of values along the regression line compared to Random Forest. This effect is particularly pronounced at higher assimilation rates (>15 µmol/(m²s)), where Random Forest exhibits systematic deviations. XGBoost’s improved accuracy and robustness make it the preferred model for photosynthesis prediction (Burdett and Wellen, 2022; Ha et al., 2024; Li et al., 2021; Singh et al., 2023; Zhang et al., 2020).

### Evaluation of our model

While the model demonstrates high predictive power (R² = 0.847), limitations remain. Studies by Ogren and Sjöström (1990) and Sharma et al. (2015) indicate that Fv/Fm correlates well with quantum yield under low to moderate light conditions (220 PAR – 500 PAR (Ogren and Sjöström, 1990; Sharma et al., 2015). Lakowicz (2006) further demonstrated that fluorescence intensity reflects photosynthesis quantum yield and chlorophyll concentration (Lakowicz, 2006). This correlation is particularly strong in C4 plants due to their reduced photorespiration.

However, Bucher et al. (2018) found that Fv/Fm was not a reliable predictor of assimilation rates in field conditions (Bucher et al., 2018). This discrepancy may stem from the influence of competing electron-consuming processes, such as photorespiration, nitrogen metabolism, the Mehler reaction, and oxygen photoreduction, which are not directly accounted for in fluorescence-based measurements (Heber, 2002; Kalaji et al., 2017; Maxwell and Johnson, 2000).

Another limitation arises from the depth of PAM fluorescence measurements, which only capture the outermost leaf layers. If measurements are restricted to the adaxial leaf surface, data reflect only chloroplast activity within the palisade parenchyma, neglecting potential differences in spongy mesophyll layers (Troeng and Linder, 1982).

Furthermore, photosynthesis and electron transport rates fluctuate seasonally within a species (Holland et al., 2014; Lazár, 2006; Neuner and Pramsohler, 2006; Oquist and Huner, 2003). These temporal variations must be considered in future model refinements to enhance predictive accuracy under natural conditions. Despite these limitations, our findings provide a powerful tool for further optimizing photosynthesis modeling using machine learning.

The integration of machine learning techniques into photosynthesis modeling offers promising perspectives for future research. A particularly valuable direction could involve combining mechanistic models with machine learning approaches to leverage the strengths of both. Specifically, the development of hybrid models that integrate the physico-chemical foundations of photosynthesis with the pattern-recognition capabilities of machine learning algorithms would allow for the analysis of complex relationships within large datasets.

## Conclusion

The study demonstrates a significant advancement in data-driven prediction of photosynthetic assimilation rates using machine learning. We identified a biologically consistent hierarchy of predictors. The results validate the selection of features and their alignment with fundamental biological processes. The XGBoost algorithm demonstrated superior accuracy and reliability compared to Random Forest, particularly in managing difficult relationships and interactions between features. This highlights the potential of machine learning in advancing our understanding of photosynthetic processes and paving the way for further integration of data-driven approaches into plant science. In future applications, such models could support precision agriculture by enabling rapid, non-invasive monitoring of plant performance under field conditions. Especially under abiotic stress scenarios such as drought or high temperatures, this approach may help to identify physiological limitations and optimize crop management strategies.

## Data Availability

The datasets presented in this study can be found in online repositories. The names of the repository/repositories and accession number(s) can be found below: https://github.com/KlirS/Model-for-PAM-Fluorescence-Gas-Exchange-Correlation.
